# Subjective and Cardiovascular Responses to an Acute Laboratory Gambling Task in Men and Women

**DOI:** 10.3389/fpsyt.2022.702298

**Published:** 2022-06-06

**Authors:** Louise Miller, Anna Söderpalm Gordh

**Affiliations:** ^1^Addiction Biology Unit, Department of Psychiatry and Neurochemistry, Institute of Neuroscience and Physiology, The Sahlgrenska Academy at University of Gothenburg, Gothenburg, Sweden; ^2^Department of Addiction and Dependency, Sahlgrenska University Hospital, Gothenburg, Sweden

**Keywords:** gambling, sex differences, subjective effects, slot machine, gender

## Abstract

Men have previously been overrepresented in gambling for money but in recent years there has been an increase in recognition that women who gamble are “catching up” with their male counterparts. There have been few experimental studies investigating the subjective effects of gambling, and even less have explored the gender differences. As gender differences previously have been reported in the subjective effects of several drugs of abuse such as opioids, amphetamines and alcohol, we sought to investigate if the subjective effects of gambling also differed by gender. The present article analyzes if gender modulates the subjective and physiological effects of an acute laboratory gambling task in healthy men and women. Eighty-two men and women (*n* = 35 men, *n* = 47 women) were tested with an online slot machine gambling session and self-report questionnaires of mood and blood pressure were taken before and after gambling. Both men and women showed stimulatory effects of gambling i.e., feelings of high and euphoria and but no differences were found between genders. Findings suggest that both men and women equally experience a pattern of stimulatory effects of gambling from the gambling situation. Gambling therefore seems to have the same abuse potential in both men and women. Although the gap between men and women is narrowing, immediate subjective and physiologic responses do not explain gender differences in the epidemiology of pathological gambling. The contexts and factors that foster or hinder the evolution of gambling addiction in males and females should be further explored. This conclusion is interesting in light of that men are over three times more at risk to experience gambling related problems than women and this risk may depend on other factors involved in the development of addiction.

## Introduction

Men have a higher prevalence of problem- and pathological gambling than women do. In Sweden it is estimated that 2.5% of women and 6.3% of men have a problem with gambling (1). In a British sample of over 1,000 treatment seeking pathological gamblers it was demonstrated that 92.5% were men and 7.5% were women (2), and in the last few decades there has been a substantial increase in female gambling in several countries worldwide [for review see (3)]. Prevalence studies from New Zealand (4), Australia (5), Britain (6), Canada (7), Finland (8) and Sweden (9, 10) all demonstrate an increased frequency in female gambling. Though the gap is narrowing men still show a higher gambling participation, gambling expenditure and prevalence of gambling than women do [for review see (11)].

One explanation for the narrowing gap may be that online gambling has increased significantly in recent years. It has been seen that both men and women prefer online gambling but women specifically choose this method. In a recent sample of 7,463 problem gamblers calling a helpline, it was seen that online gambling was associated with the highest problem severity level and that 86% of the women stated that they had gambled using online games during the previous month (12). As a consequence, proportions of online gamblers seeking treatment at addiction clinics has increased. In a Swedish study it was seen that as many as 89% of gamblers seeking treatment reported that online gambling lead to their gambling problems (13). Online gambling, in particular slot machine play, has further been cited as the most problematic form of gambling. Due to the high rates of near misses and usage of stop buttons, the availability and fast speed between bets and wins, online gambling are more rewarding than other games (14–16).

Further, increased attention has been paid to co-occurring substance use disorders in relation to gambling disorder [for review see (17–19)] but knowledge about gender specific patterns are lacking. In a study by Sundqvist and Rosendahl (20) they found that alcohol problems are also strongly associated with gambling in both women and men, with a three times higher risk compared to the controls. They also found that women more often initiated gambling in relation to a problem with substances while men did the opposite. Men initiated gambling more often without the concurrence of any psychiatric or substance abuse problems. The knowledge about connections between substance use disorders and gambling is interesting in the light of previous research on the subjective effects of alcohol in where it is well-known that initial stimulant effects of alcohol predict heavy alcohol use longitudinally. In a recent study by King et al. (21), 190 healthy drinkers were challenged with alcohol and their stimulant responses to alcohol predicted heavy alcohol use in both a 6 and 10 year follow up. The stimulant responses to alcohol were also increased in magnitude over a 10 year period in those individuals who developed an alcohol use disorder (21). Such a finding demonstrates that the idea and the methodology, that changes in subjective responses to a drug measured by self-reported questionnaires, is a valid, up to date and an accurate method for studying the effects of a gambling task in healthy volunteers.

Little attention has been paid to the subjective effects produced by gambling even though it is well-known that both men and women have been found to respond differently to the effects of several different drugs. For example, men have showed increased feelings of self-reported subjective high and stimulation when exposed to cocaine, d-amphetamine and alcohol compared to women (22–24). In addition, contradictory results has been seen in two Positron Emission Tomography studies in where in one of them a greater d-amphetamine-induced dopamine release was seen in women compared to men (25) and in the other study there was a lack of gender difference in d-amphetamine-induced dopamine release (26). These drugs are well-known to produce stimulation through the brain dopamine system in both humans and animals. Women on the other hand have been found to report more distinct unpleasant effects of opioids (27) and greater negative effects after morphine administration than men (28), drugs known to be mediated by the endogenous opioid system. Women have also been found to show increased self-reported sedation in response to methamphetamine compared to men (29). It has also been recognized that gonadal hormones causes a gender difference in response to pharmacological drugs such as psychostimulants (22, 30, 31). The subjective effects of cocaine have for example been found to be more or less pleasurable depending on the menstrual phase (22, 31).

Based on these studies there are reasons to believe that there also may be gender differences in response to self-reported effects of gambling i.e., that men may experience increased stimulative effects in response to gambling.

Similarly, as to other drugs of abuse, the literature describes underlying neurobiological mechanisms such as the monoamine systems (e.g., dopamine) and demonstrate increased dopamine levels in response to gambling situations. For example, Zack and Poulos (32) tested a dopamine D2 antagonist, Haloperidol, in responses to a gambling task on a slot machine in the laboratory during 15 min in pathological gamblers and controls. They found that Haloperidol significantly increased blood pressure and self-reported rewarding stimulative effects on scales specifically developed to measure drug effects after gambling in pathological gamblers. Further, in an *in vivo* dopamine release study, subjects playing videogames in the laboratory showed increased dopamine release comparable to the dopamine levels induced by psychostimulants drugs such as amphetamines (33). Earlier research has also demonstrated that gambling can stimulate an amphetamine (AMPH) like effects. Zack and Poulos found that AMPH primed motivation to gamble and that the severity of problem gambling predicted the AMPH-induced motivation (34, 35). Taken together, these studies suggest that the subjective effects of gambling share similar stimulative properties as psychostimulants probably due to the involvement of catecholamine's.

Both pathological and regular gamblers, have also previously shown increased subjective arousal and stimulation in response to gambling [for review see (36)]. In a study with the attempt to identify mood state critical to gambling maintenance it was found that regular and pathological gamblers experienced significantly more excitement after playing on a slot machine, as decided through a face to face interview with participants coming out from an amusement arcade (37). Positive subjective arousal has also been found to increase to a greater extent in problem gamblers than in a group of non-problem gamblers in response to both wins and losses in an electronic gambling machine (38). Arousal in these subjects was established before and after gambling measured by the Spielberger's State Trait Anxiety Inventory (39), an instrument used to diagnose anxiety and to distinguish it from depressive syndromes through five valence items such as pleasant, joyful, self-confident, upset, and regretful. Further, studying gender differences and the role of winning, both men and women were exposed to a slot machine game finding that both genders reported increased heart rate and arousal to the same extent in relation to a winning situation (40). Arousal was measured by a four item subscale from the Speilberger State–Trait Anxiety Questionnaire (39) and by the Sensation Seeking Scale (41). None of these instruments are developed and validated to measure drug effects.

Similar results were obtained in Sharpe (42), finding that problem gamblers became autonomically aroused i.e., skin conductance levels when they were asked to recall a gambling winning situation in comparison to social gamblers but no differences was found when the groups were asked to recall a loosing situation. In addition, a study with only women with the purpose to describe the physiological responses occurring during slot machine play with or without monetary stakes it was found that blood pressure, heart rate, respiratory rate, skin conductance, and skin temperature rose during gambling and fell during recovery. This study showed that women find slot machine play physiologically arousing (43). These studies suggests that gambling share arousal-like effects similar to those of drugs of abuse i.e., excitement, joy and physical arousal even though the measurements lack evidence to measure the effects of drugs. With these experimental studies in mind we exposed recreational high, low and non-gamblers to a casino laboratory gambling task. We found that gamblers and more specifically high recreational gamblers, who gambled around three hours a week, showed increased self-reported subjective high and stimulation in comparison to non-gamblers after having gambled on a slot machine for 10 min (44). Although both men and women were included in these studies, only the study of Coventry and Hudson (40) was designed to study differences between the genders.

Gambling seems to trigger states of both physiological and subjective arousal which in turn may be linked to a path into gambling addiction. An established pathway model into a gambling addiction is called the “antisocial impulsivist problem gamblers” in where more men than women belong to Blaszczynski and Nower (45). In this pathway a subgroup of gamblers is referred to as “action seekers” in where arousal from gambling is a necessary component for the continuation of gambling (36). This subgroup may very well be overrepresented by individuals showing a heighted physiological and subjective responses to a gambling task. There is also an interesting link between the subgroup “action seekers” and substance use disorder in where it is well-established that increased physiological and subjective responses is a predictor for an alcohol use disorder (21).

Based on men's higher prevalence of problem gambling than women, men's sensitivity to other drugs of abuse that produce euphoria and stimulation, men's pathways through excitement and arousal into gambling problems and that gambling produces stimulation comparable to a psychostimulant. The outlined studies are therefore building evidence for our hypothesis that men will experience a gambling task more stimulating than women do. To our knowledge, there are no other studies that have challenged healthy volunteers in the laboratory with an online gambling task measuring self-reported drug effects.

## Methods

### Subject Recruitment and Screening

This study is a *post-hoc* analysis of a previously published study (44) in which we found that high recreational gamblers reported stimulation in response to a short gambling task. Eighty-two healthy men (*n* = 35) and women (*n* = 47) recruited via advertisements were initially screened by telephone for major eligibility criteria. Participants were invited to the laboratory for further screening upon meeting the inclusion requirements of: age (19–65), normal BMI (18.5–25), moderate consuming of alcohol (i.e., no more than 9 standard drinks per week for women and 12–14 for men), negative history of substance abuse and or negative history of somatic diseases. Subjects completed the DSM-IV diagnoses for pathological gambling (46) and did not currently receive treatment for gambling addiction. They also answered questions about time in minutes that they spent gambling each week, and on average, how much money they would have spent on a regular day gambling within the last 3 months. Alcohol Use Disorders Identification Test [AUDIT (47)] and the psychiatric symptom checklist [SCL-90 (48)] assessing medical and psychiatric histories. The study was approved by the regional ethics committee of the University of Gothenburg and complied with the guidelines of the Declaration of Helsinki.

### Design and Procedure

The study was conducted in a comfortable environment, furnished like an apartment living room (see section laboratory setting). Subjects were always run alone. The session procedure was as follows: Subjects arrived at the laboratory between 9 am and 4 pm. Objective measures of blood pressure (BP) and subjective self-report measures of the Drug Effects Questionnaire (DEQ) (49) and the Addiction Research Inventory Scale (ARCI) (50) were assessed at baseline (0 min) with questionnaires printed out in paper and filled out in ink pen. Participants were then asked to virtually play on an online casino gambling program on a computer for 10 min until an alarm sounded which indicated the end of the gambling period. This is what we call a gambling task. This 10 min period is what we will be referring to as the “gambling task” in the following. The participants were unaware of the length of time for which they gambled. Directly after playing on the slot machine the last measures of the Drug Effects Questionnaire (49), ARCI and the objective measures were taken. At the end of the study, participants were debriefed by the experimenter and received compensation for their participation.

### Gambling Model

Each participant was presented with the online casino All JSlots, version 2.2. on a laptop during a 10 min period. This slot machine mimics available online versions and is complete with all reel features. The specific feature with the slot machine is that wins are positively reinforced via instrumental conditioning, which facilitates the development of problem gambling. It is possible that wins are made more capable of attracting attention or being processed (at a conscious level) by co-administration of flashing lights and music relative to losses that typically do not include these sensory events i.e., that classical conditioning amplifies the rewarding properties of the monetary payoff itself. Jackpot wins and losses, flashing sequences and music were accompanied with line wins, along with brightly colored imagines of a mix of cherry's, oranges, motorcycles and various other images. The starting point was a credit of SEK 75 (1 credit = SEK 1), and every spin on the slot-machine simulation let the participant gamble for 1, 2, or 3 credits and is consistent as every bet is a timed reel feature. The screen displays the “payline,” total credits, bet and amount that the winner can be paid out. This simulates online slot machines, as participants were able to decide for themselves if they would like to bet one, two or three credits, which represented the number of lines played. All Jslots line credits represents the number of lines played. One credit equals one line and two, two lines and so forth. This in turn increased chances of winning lines and a potential pay-out. Participants played on average 55.5 (6.8) M (SEM) spins during the 10-min period. The game was randomized and each person achieved a different amount of “wins or losses” similar to a real online slot machine. Subjects played only for slot machine credits, which were not paid out in cash. It has specifically been found that slot machine play increases positive emotions and the excitement of playing which may lead people to gamble more (51).

### Laboratory Environment

The study was conducted in a laboratory setting at Sahlgrenska University Hospital in Gothenburg Sweden as part of the Addiction Biology Unit (ABU), Section for Psychiatry and Neurochemistry, Institute of Neuroscience and Physiology. The laboratory setting was designed to look and feel like a living room to simulate how people would online gamble at home. The room comprised of a large sofa, table, armchairs, curtains, bookshelf, paintings on the wall, kettle and magazines. Participants were asked not to work, study or use their phones. When they had finished their paperwork, they were told to relax and wait for the next step in the process of the study.

### Dependent Measures

In the present study the participants received a controlled gambling task under laboratory conditions. We therefore are able to document a participant's acute experience with gambling. The dependent measure was the Drug Effect Questionnaire (DEQ), the Addictions Research Center Inventory (ARCI) and the physiological measures were heart rate and blood pressure.

### Self-Reported and Objective Measures

Participants were exposed to standardized questionnaires primarily used for drug abuse. All questionnaires were used in a version translated to Swedish. The questionnaires used to assess mood states and subjective drug effects described below are sensitive to the effects of a variety of psychoactive drugs [e.g., (52, 53)]. Research on the subjective responses to drugs provides valuable information about individual differences in responses to a variety of drugs. Responses such as euphoria and liking are considered to be related to frequency of use and abuse [for review see (54)]. One of the most common measures are the Drug Effects Questionnaire (DEQ) described below and it assesses two key components of a subjective response, the strength of an effect and the desirability of the effects (54). DEQ is widely used and has demonstrated utility assessing drug effects of different drugs in laboratory settings (55). The second measure used is the Addiction Research Center Inventory (ARCI) described below. The ARCI scale has extensively been used in research to take repeated measures of subjective effects of a variety of drugs (56). The ARCI has also been proven sensitive to the effects of gambling (44, 57).

### The Drug Effect Questionnaire

Drug Effect Questionnaire (DEQ) assesses the extent to which subjects experience drug effects (49). The DEQ is widely used as a screening method for drug abuse and risk of abuse. It allows the participant to evaluate the effect of a specific drug using four sub-categories: “effect” (do you feel an effect? None at all to A lot), “like” (do you like the effect? Not at all to Like very much), “high” (do you feel a high? Not at all to Very much), and “want more” (do you want more? Not at all to Very much). The DEQ versions used in this study employed a 100 mm visual analog scale (VAS) anchored by “not at all” and variants of “extremely” to capture the post-drug experience of “effect,” “like,” “high,” and “want more.” However, how well and to what extent the DEQ response corresponds to a drug response outside the laboratory is not well-studied (54).

The Addiction Center Research Inventory (ARCI) (50, 56). The ARCI is a 49-item true-false questionnaire designed to assess subjective responses to different categories of abused drugs. It consists of five subscales: Amphetamine-like effects (A e.g., increased energy, sense of well-being), Benzedrine-like effects (BG e.g., increased energy, intellectual productivity), Morphine-Benzedrine-like effects (MBG e.g., pleasant somatic experiences, euphoria), Lysergic Acid Diethylamide-like effects (LSD e.g., dysphoria, somatic discomfort), and Pentobarbital-Chlorpromazine-Alcohol-like effects (PCAG e.g., sedation, psychomotor retardation). The true and false questions are scored with one point given for each response that corresponds with the direction of the scale. ARCI was used to measure subjective responses to gambling and we focused our analyses on the Benzedrine Group (euphoric effects) and Amphetamine-like (stimulant effects scales) as these represent the typical positive, rewarding effects of amphetamine. Reviews that summarize evidence that these scales are sensitive to the acute effects of amphetamine and that they are predictive of amphetamine choice (53, 54, 58).

### Physiological Measures

#### Heart Rate and Blood Pressure

A *Dynamap*^®^ monitor was used to monitor heart rate and blood pressure. Measurements were taken prior to and directly after slot-machine gambling, to ensure any fluctuations were recorded in response to the gambling task, as seen in previous research (see introduction).

### Statistical Analyses

#### General Description of the Data Analysis

For descriptive purpose mean and standard error of mean (SEM) were presented for continuous variables. The analysis was performed using IBM Statistical Package for Social Sciences software version 25.0 [SPSS, Inc., (59)]. Data was analyzed using mixed models, response profile analysis. Data was analyzed using *t*-tests, one way ANOVA and by using General Linear Models, multivariate ANOVAs. For all analyses and comparisons, statistically significant *p*-values were set at <0.05 and corrections for multiple comparisons were made with a Bonferroni test. Primary outcome measures were the “effect,” “like,” “high,” and “want more” scales from the DEQ. In addition, we focused on the stimulant (A scale) and euphoria (MBG) subscales of the ARCI. These scales represent the typical rewarding and hedonic mood effects of drugs. For our hypothesis, males (gambling time 75 ± 40 min/week) were compared to females (gambling time 22 ± 13 min/week). We did three types of analyses. In the first analysis we tested if the demographic variables differed between men and women. In the second analysis we tested for baseline differences between men and women, in the third analysis we tested if there were any differences between men and women in response to the gambling task. Below we describe each analysis in detail.

#### Demographic Differences Between Men and Women

In the first analysis in order to test if the demographic variables differed between men and women a *T*-test was performed on gambling time, gaming time, age and total AUDIT scores.

#### Baseline Differences

In order to investigate if men and women differed at baseline on the subjective and cardiovascular measures a one-way ANOVA was performed to test differences between the two groups (men and women) on the variables A, MBG, LSD, BG, PCAG on the ARCI-scale and blood pressure (systolic, diastolic and pulse). No baseline difference between genders were tested for the DEQ since baseline was zero. The answers to the questions, do you feel the effect, do you like the effect, do you feel high, do you want more, is a zero-value at baseline. The DEQ is only presented post gambling task since the questions (effect, high, like and want more) are not valid pre-test due to the question format of “do you feel an effect” of the gambling task [see (44)].

#### Subjective and Objective Effects in Men and Women After the Online Gambling Task

A General Linear Model, a multivariate analysis with Gender × Time Spent Gambling (hi, low, no) as between subject factors and Time (pre- to post-test) as repeated measure of the dependent variables DEQ, ARCI, and blood pressure, was performed. We included Time Spent Gambling as a second between subject factor in the model since to what extent to which the DEQ, the ARCI and blood pressure increase or decreased after the 10 min gambling task do not only depend on gender but also on each participants gambling time outside the laboratory (44). The outcome may also vary dependent on the participant's age and total score on AUDIT whilst they are included as covariates in the model. First we wanted to know if there were any “acute subjective and cardiovascular effects of the gambling task in the whole group” and then we wanted to know, as our hypothesis stated, if “men differed from women on the subjective and objective measures after the online gambling task.”

## Results

### Subject Demographics

The demographic characteristics, drug use and gambling data between men and women are shown in Table 1. The mean age of the men was 28.7 (1.6) years old and females were 24.7 (0.8) years of age. The mean weight in the men was 72.9 (2.4) kg and females 64.2 (1.6) kg. The majority of the subjects were Caucasian, and a limited number were Hispanic and Asian. Males gambled for 75 (42) min/week and females gambled for 22 (13) min/week. Males gambled significantly more minutes than women did each week (*p* < 0.05), men were gaming significantly more minutes than women did (*p* < 0.05), and the men were also older than women with about 4 years (*p* < 0.01). The groups did not differ significantly on any other of the other demographic or drug use variables obtained.

**Table 1:** Demographics and drug use data between men and women.

**Gender (*n*)**	**Female (47)**	**Male (35)**
Age*	24.7 (0.8)	28.7 (1.6)
Weight (kg)	64.2 (1.6)	72.9 (2.4)
Length (cm)	167.8 (1.1)	180.9 (1.0)
**Race/ethnicity (** ***n*** **)**		
Caucasian	44	33
Asian	1	1
Hispanic	1	1
Other	1	0
**Education (** ***n*** **)**		
High School grad or less	5	0
College student	19	20
College graduate	20	13
**Current drug use**		
AUDIT (total points)	4.5 (0.4)	6.6 (0.6)
Alcoholic drinks (*n*/week)	2.7 (0.5)	4.6 (0.8)
Caffeine consumers (*n*)	27	27
Cups of coffee	7.9 (1.4)	11.8 (1.8)
Cigarette consumers (*n*/day)	3	2
Cigarettes/day	0.3 (0.2)	2.2 (1.8)
Lifetime drug use (*n* ever used)	0	0
Stimulants	0	0
Tranquilizers	0	0
Hallucinogens	0	0
Opiates	0	0
Marijuana	0	0
**Gambling**		
Participants gamble (last 3 month)	27	30
Minutes/week gambling*	22 (13.0)	75 (40)
Minutes/week computer gaming*	12 (0)	73 (22)
DSM-IV (total points)	0.10 (.06)	0.42 (.22)

*Data is presented as means and standard error of means M (SEM) and frequency (n). The significant differences were denoted with an asterisk p < 0.05*.

### Subjective and Objective Effects of Gambling in Men and Women

First, a one way between subjects ANOVA was conducted to compare the baseline conditions between men (*n* = 35) and women (*n* = 47) before the gambling task on the main dependent variables DEQ “effect,” “high,” “like” and “want more” no data shown since baseline was set to zero; ARCI-A *F*_(1, 81)_ = 3.3, *p* > 0.07, MBG *F*_(1, 81)_ = 0.39, *p* > 0.54, LSD *F*_(1, 81)_ = 0.40, *p* > 0.52, BG *F*_(1, 81)_ = 0.70, *p* > 0.41, PCAG *F*_(1, 81)_ = 0.18, *p* > 0.67, systolic blood pressure *F*_(1, 81)_ = 15.86, *p* < 0.001; diastolic blood pressure *F*_(1, 81)_ = 3.45, *p* > 0.07; pulse *F*_(1, 81)_ = 0.03, *p* > 0.87. It was found that men had a higher baseline systolic blood pressure than women (*p* < 0.001). No other statistical significant differences were found between any of the other measures at baseline. It is important to note that the two groups did not differ at baseline at any (except systolic blood pressure) of the main measures. Table 2 shows means ± SEM and Table 3 shows confidence intervals of the differences on the whole group, men and women.

**Table 2:** Subjective and objective effects between men and women after the online gambling task.

**Dependent measures M (SEM)**	**Whole group (*****n*** **=** **82)**			**Females (*****n*** **=** **47)**			**Males (*****n*** **=** **35)**		
**DEQ**	Pre	Post	Diff	Pre	Post	Diff	Pre	Post	Diff
Effect	0.0 (0.0)	30.6 (2.7)	30.6 (2.7)^*^	0.0 (0.0)	30.4 (3.6)	30.4 ± 3.6	0.0 (0.0)	34.0 (4.4)	34.0 (4.4)
Like	0.0 (0.0)	37.8 (3.1)	37.8 (3.1)^*^	0.0 (0.0)	37.1 (4.1)	37.1 ± 4.1	0.0 (0.0)	38.6 (5.0)	38.6 (5.0)
High	0.0 (0.0)	19.3 (2.5)	19.3 (2.5)^*^	0.0 (0.0)	21.1 (4.1)	21.1 ± 4.1	0.0 (0.0)	18.6 (4.1)	18.6 (4.1)
Want more	0.0 (0.0)	21.1 (2.5)	21.1 (2.5)^*^	0.0 (0.0)	23.6 (3.0)	23.6 ± 3.0	0.0 (0.0)	17.8 (4.0)	17.8 (4.0)
**ARCI**									
Amphetamine	3.6 (0.2)	3.7 (0.2)	−0.05 (0.2)	3.3 (0.3)	3.7 (0.4)	0.4 (0.1)	4.1 (0.3)	3.7 (0.4)	−0.4 (−0.1)
Morphine	5.5 (0.4)	5.2 (0.3)	0.04 (0.3)	5.2 (0.4)	5.3 (0.5)	0.1 (0.1)	5.3 (0.7)	5.2 (0.5)	−0.1 (0.2)
LSD	3.3 (0.2)	3.1 (0.2)	0.16 (0.1)	3.1 (0.2)	3.2 (0.3)	0.1 (0.1)	3.4 (0.6)	3.0 (0.3)	0.4 (0.3)
Benzedrine	3.7 (0.2)	3.9 (0.2)	−0.17 (0.2)	3.6 (0.2)	3.6 (0.4)	0.0 (0.2)	3.9 (0.4)	4.1 (0.3)	0.2 (0.1)
Pent-Alk	3.70 (0.2)	3.9 (0.2)	−0.18 (0.2)	3.5 (0.3)	3.8 (0.4)	0.3 (0.1)	3.9 (0.5)	3.8 (0.4)	0.1 (0.1)
**BP**									
Systolic	121.3 (1.5)	117.5 (1.5)	5.37 (2.0)^*^	116.9 (1.8)^*^	113.3 (1.9)	−3.6 (−0.1)	127.7 (2.3)^*^	123.4 (2.1)	−4.3 (−0.2)
Diastolic	78.2 (1.0)	75.5 (0.9)	3.63 (1.3)^*^	76.9 (1.4)	74.7 (1.3)	−2.2 (−0.1)	80.0 (1.6)	76.5 (1.5)	−3.5 (−0.1)
Pulse	71.8 (1.1)	69.0 (1.0)	4.49 (1.4)^*^	72.1 (1.4)	69.5 (1.3)	−2.6 (−0.1)	71.5 (2.0)	68.4 (1.9)	−3.1 (−0.1)

*Data is presented as means and standard error of means (SEM)*.

^*^denotes a statistical difference.

**Table 3:** Confidence Intervals are presented as pre-test, post-test, and difference score for all dependent measures taken in the study in data on whole group, women and men.

**Dependent measures [95% CI]**	**Whole group (*****n*** **=** **82)**			**Women (*****n*** **=** **47)**			**Men (*****n*** **=** **35)**		
**DEQ**	Pre	Post	Diff	Pre	Post	Diff	Pre	Post	Diff
Effect	0.0 (0.0)	25.3–36.0	25.3–36.0	0.0 (0.0)	23.1–36.5	23.1–36.5	0.0 (0.0)	22.8–40.6	22.8–40.6
Like	0.0 (0.0)	31.5–44.0	31.5–44.0	0.0 (0.0)	29.6–46.0	29.6–46.0	0.0 (0.0)	23.9–47.6	23.9–47.6
High	0.0 (0.0)	14.3–24.3	4.3–24.3	0.0 (0.0)	13.6–27.4	13.6–27.4	0.0 (0.0)	10.4–24.9	10.4–24.9
Want more	0.0 (0.0)	16.0–26.2	16.0–26.2	0.0 (0.0)	16.7–31.1	16.7–31.1	0.0 (0.0)	10.7–23.9	10.7–23.9
**ARCI**									
Amphetamine	3.2–4.1	2.7–3.5	−0.5–0.6	2.7–3.9	3.0–4.3	−0.3–1.1	43.4–4.9	2.9–4.6	−0.4–1.2
Morphine	4.6–6.0	4.5–6.0	−0.6–0.7	4.2–6.0	4.7–6.2	−0.6–1.0	4.4–6.7	4.1–6.3	−0.8–1.5
LSD	2.9–3.7	2.7–3.5	−0.2–0.5	2.8–3.6	2.7–3.9	−0.5–0.6	2.8–4.1	2.5–3.5	−0.1–1.0
Benzedrine	3.3–4.1	3.5–4.3	−0.2–0.5	3.0–4.1	3.1–4.2	−0.4–0.6	3.2–4.6	3.5–4.9	−0.2–0.8
Pent-Alk	3.2–4.2	3.3–4.4	−0.3–0.7	2.9–4.2	3.3–4.6	−0.2–1.0	3.0–4.6	2.9–4.6	−0.7–0.8
**BP**									
Systolic	118.3–124.4	114.5–120.5	1.3–9.4	112.9–120.1	109.3–116.9	−0.3–7.0	123.6–132.4	119.4–127.6	−0.1–16.1
Diastolic	76.2–80.3	33.6–77.5	1.1–6.6	74.0–79.2	71.8–76.8	−0.1–4.7	77.2–83.7	74.1–80.2	0.5–10.4
Pulse	69.7–74.1	67.1–71.1	4.1.7–7.2	69.3–74.7	67.3–71.8	0.3–4.7	68.0–75.3	64.8–72.0	1.5–12.8

### Subjective and Objective Effects in Both Men and Women as a Whole Group After the Online Gambling Task

**Figure 1:**
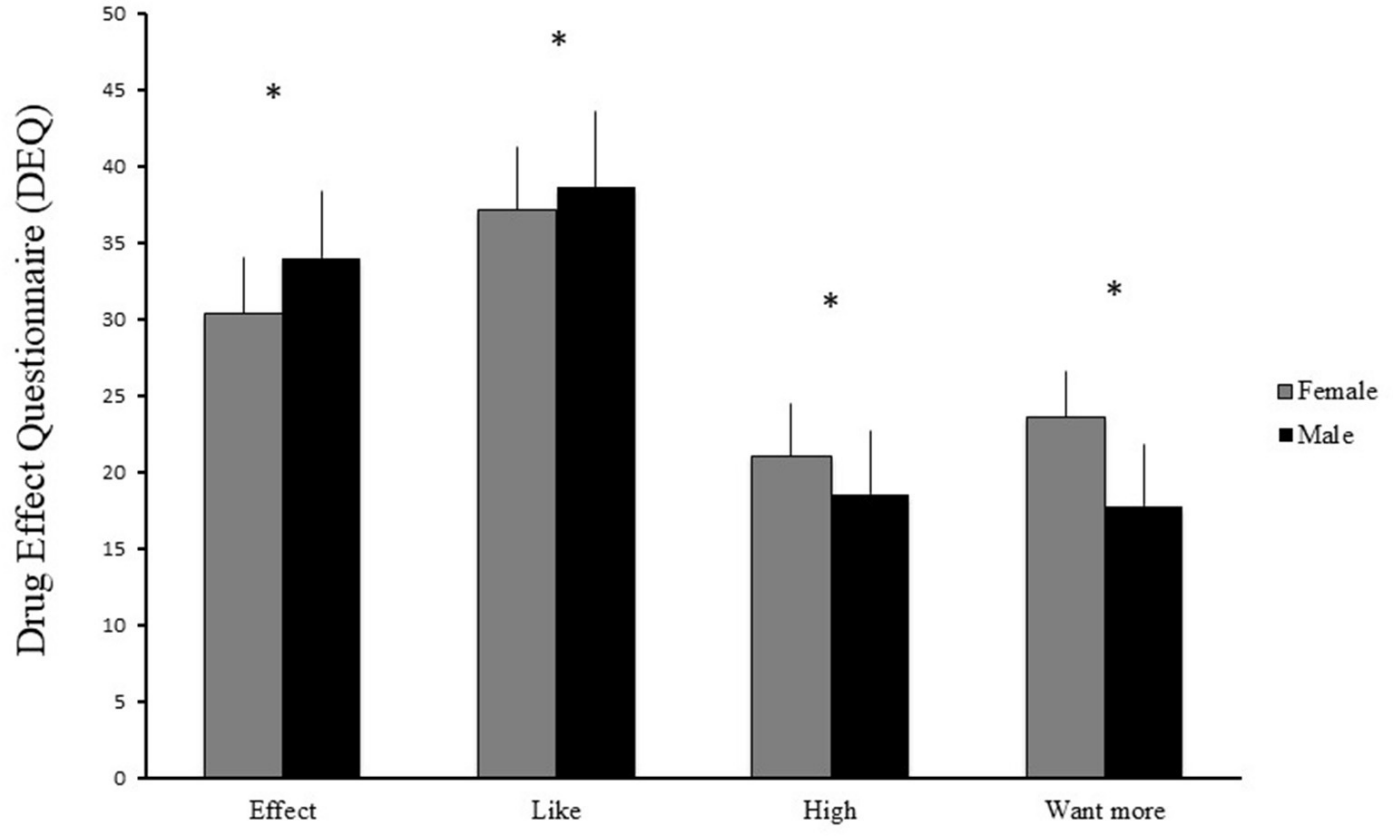
Mean difference scores and standard error of mean (SEM) on the Drug Effects Questionnaire (DEQ) between pre and post-test for Effect, High, Like, and Want more after the 10 min online slot machine task between men (black bars) and the women (gray bars). The asterisks denote significant differences between pre and post-test (*p* < 0.05) for the data of the whole group.

**Figure 2:**
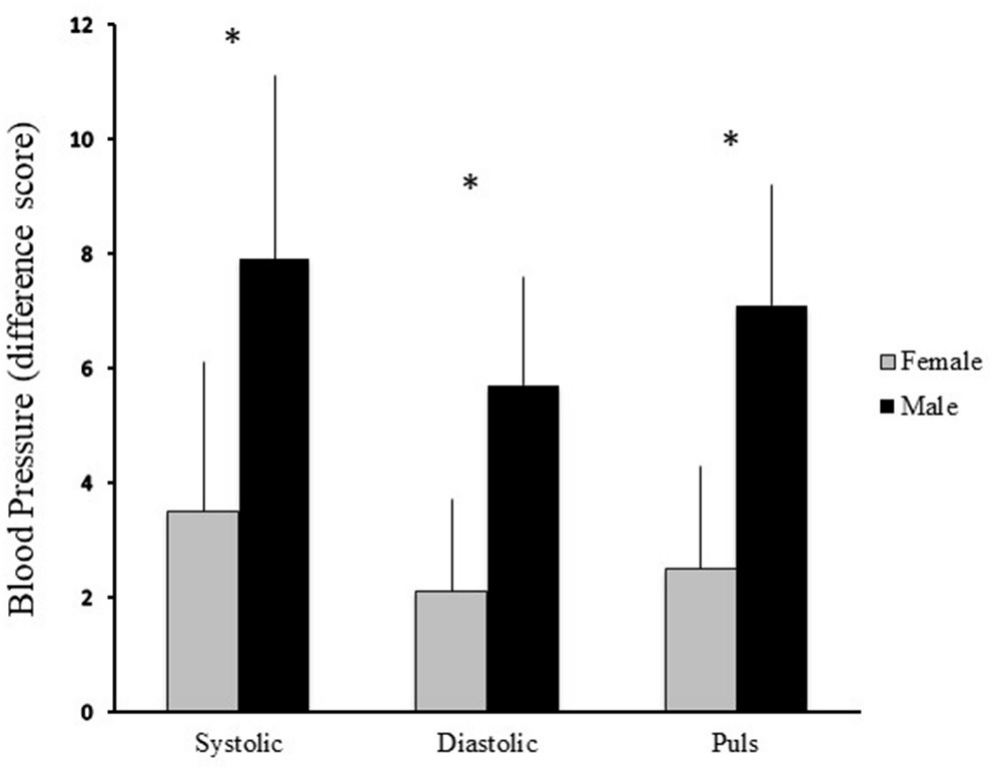
Mean difference scores and Standard Error of Mean (SEM) on the blood pressure between pre and post-test for systolic, diastolic and pulse after the 10 min online slot machine task between men (black bars) and the women (gray bars). The asterisks denote significant differences between pre and post-test (*p* < 0.05) for the data of the whole group.

First we wanted to know if there were any acute subjective and cardiovascular effects of the gambling task in the whole group. In the GLM, in a multivariate test, with Gender and Time Spent Gambling as between subject factors and Time (pre to post) on the dependent variables we found that the gambling task produced significant main effects on the DEQ in the whole group (Figure 1). This effect was not seen on the ARCI scale or in Blood Pressure (Figure 2). It was found that the DEQ increased “effect” *F*_(1, 72)_ = 25.6, *p* < 0.001, “like” *F*_(1, 69)_ = 15.1, *p* < 0.001, “high” *F*_(1, 72)_ = 14.52, *p* < 0.001 and “want more” *F*_(1, 72)_ = 7.77, *p* < 0.007 after the gambling task. The ARCI scale did not have a significant effect in the total group, ARCI-A *F*_(1, 73)_ = 0.25, *p* > 0.62, MBG *F*_(1, 73)_ = 0.67, *p* > 0.41, LSD *F*_(1, 73)_ = 0.50, *p* > 0.48, BG *F*_(1, 73)_ = 0.04, *p* > 0.84 and PCAG *F*_(1, 73)_ = 1.03, *p* > 0.31. The analysis on blood pressure also showed a non-significant result in the total group in diastolic *F*_(1, 72)_ = 1.39, *p* > 0.24, systolic *F*_(1, 72)_ = 0.06, *p* > 0.80 and in the measure of pulse *F*_(1, 71)_ = 1.10, *p* > 0.29.

### Subjective and Objective Effects Between Men and Women After the Online Gambling Task

Second, as our hypothesis stated, we wanted to know if men would show increased subjective effects after a gambling slot machine task in comparison to women. In the same analysis we saw a non-significant result between Gender × Time Spent Gambling on the DEQ “effect” *F*_(2, 72)_ = 0.55, *p* > 0.57, Age *F*_(1, 72)_ = 0.91, *p* > 0.34, AUDIT scores *F*_(1, 72)_ = 2.71, *p* > 0.10. We found no difference between gender on “like,” *F*_(2, 69)_ = 0.40, *p* > 0.67, Age *F*_(1, 69)_ = 0.75, *p* > 0.39 and AUDIT scores *F*_(1, 69)_ = 0.01, *p* > 0.90. No significant difference was seen on “high” *F*_(2, 72)_ = 0.09, *p* > 0.91, Age *F*_(1, 72)_ = 1.10, *p* > 0.29 or AUDIT scores *F*_(1, 72)_ = 1.71, *p* > 0.19 or on “want more” *F*_(2, 72)_ = 0.29, *p* > 0.74, Age *F*_(1, 72)_ = 0.48, *p* > 0.49 and AUDIT scores *F*_(1, 72)_ = 0.03, *p* > 0.86.

In the same analysis we also saw a non-significant effect on the “ARCI-A” *F*_(2, 73)_ = 0.49, *p* > 0.61, Age *F*_(1, 73)_ = 0.17, *p* > 0.67 and AUDIT scores *F*_(1, 73)_ = 0.13, *p* > 0.71. On the “MBG” we found no significant effects *F*_(2, 73)_ = 0.83, *p* > 0.43, Age *F*_(1, 73)_ = 1.00, *p* > 0.32 and AUDIT scores *F*_(1, 73)_ = 0.00, *p* > 0.95. On the “LSD” we found no significant effects between *F*_(2, 73)_ = 0.10, *p* > 0.89, Age *F*_(1, 73)_ = 1.52, *p* > 0.21 and AUDIT scores *F*_(1, 73)_ = 0.18, *p* > 0.67. Further, we saw a non-significant effect on the “BG” *F*_(2, 73)_ = 0.34, *p* > 0.71, Age *F*_(1, 73)_ = 0.35, *p* > 0.55 and AUDIT scores *F*_(1, 73)_ = 0.50, *p* > 0.47. We also saw a non-significant effect on the “PCAG” *F*_(2, 73)_ = 0.80, *p* > 0.44, Age *F*_(1, 73)_ = 1.52, *p* > 0.22 and AUDIT scores *F*_(1, 73)_ = 0.05, *p* > 0.82.

In the same analysis we found a non-significant effect on “systolic blood pressure” *F*_(2, 72)_ = 0.15, *p* > 0.85, Age *F*_(1, 72)_ = 1.72, *p* > 0.19 and AUDIT scores *F*_(1, 72)_ = 0.29, *p* > 0.58. We also did not see a significant effect on the “diastolic blood pressure” *F*_(2, 72)_ = 0.01, *p* > 0.53, Age *F*_(1, 72)_ = 1.11, *p* > 0.29 and AUDIT scores *F*_(1, 72)_ = 1.78, *p* > 0.18. At the last measure “pulse” we did not see a significant effect *F*_(2, 71)_ = 0.47, *p* > 0.62, Age *F*_(1, 71)_ = 0.29, *p* > 0.58 or AUDIT scores *F*_(1, 71)_ = 1.63, *p* > 0.20. This means that the effect of the gambling task did not differ between men and women either on the DEQ or the ARCI scale or in Blood Pressure.

At the end of the gambling session women “won” 355.5 (34.5) M (SEM) Swedish crones, USD (≈$38) and the men had won 356.3 (37.8) M (SEM) Swedish crones, USD (≈$38). There was no difference between the winning outcomes between genders.

## Discussion

The present study is a *post-hoc* analysis from a previous study. In the present study we found that both men and women as a group quite equally showed increased self-reported stimulative effects on the DEQ on all the four scales i.e., “effect,” “like,” “high,” and “want more” after an online casino gambling task in the laboratory. However, when we tested for differences between gender we found no effects on the self-reported scales (DEQ or ARCI) or Blood Pressure after the gambling task.

After the gambling task both men and women reported increased responses on the subjective measures taken in the study such as on the Drug Effects Questionnaires. The theory that the stimulant and rewarding properties of a specific drug (60, 61), or as in this case a gambling behavior, has abuse potential was supported by both the effects seen from the DEQ scale and the blood pressure in both men and women in our study. That both men and women respond in a comparable way is supported by previous research on the clinical effects of gambling. Coventry and Hudson (40) challenged 22 males and 20 females with slot machine play, finding that both genders reported increased heart rate and arousal to the same extent. Brown et al. (38) examined self-reported changes in arousal and affective valence in 26 males and 41 female problem and non-problem gamblers, playing on electronic gaming machines finding that both groups experienced increased arousal. Similar results were obtained in Sharpe (42), in 20 men and 13 women, finding that problem gamblers became autonomically aroused when they were asked to recall a gambling winning situation in comparison to social gamblers but no differences were found when the groups were asked to recall a loosing situation. One must bear in mind that only the study of Coventry and Hudson (40) were designed to study differences between the genders and therefore differences were not reported in the other studies. These results provide evidence that gambling produces both subjective effects and cardiovascular arousal in both men and women. This is similar to other drugs of abuse, probably due to the involvement of catecholamine's and is to be seen as potential predictor to continued use of gambling in real life (21).

Our results were not in line with our hypothesis in which we predicted that men would show a increased rewarding effect after the gambling task in comparison to women. A better understanding of interactions between the subjective effects and for example a reward expectancy, gambling as a socially accepted recreation and accessibility of online gambling may account for possible explanations of gender differences. Excitement generated by the expectancy of winning money has also been found to seen influence arousal. Finding shows that a high expectation of winning money leads to increased heart rate in recreational gamblers, (62) and in a fMRI study, it has been seen that problem gamblers shows higher activity in the reward system during expectation (63). Further, an association between self-reported high excitement and an increase in dopamine release during a gambling task has also been seen in pathological gamblers (64). These studies highlight that gamblers show altered reward expectation when presented with a gambling experience. Even though these studies mostly investigate males none of the studies considered gender differences. Research also suggest that it is a cultural difference with more men seeing gambling as a socially accepted recreation compared to women (65). Being a male is continuously identified as a risk factor for gambling disorder [for review see (65)] and men's personality traits i.e., men being more impulsive than women, makes them more likely than women to become problem gamblers [for review see (66)]. Men are also betting on sports more than women do and there is a strong relationship between gambling on sports and problem gambling (67). It has been known for a long time that there are gender differences in all phases of the addiction process. Men have shown to demonstrate a gambling dysfunction earlier than women do. They initiate gambling, begin to gamble regularly, try to stop gambling and enter treatment at an earlier age than women do (68–70). Men have also seen to be 2.3 times more at risk after gambling exposure and 3.6 times more likely to experience gambling related problems than women (70).

It has also been documented that there are differences in men and women's pathway to addiction. Initially for many men and women, drugs are taken for their positive reinforcing effects i.e., they produce euphoria and liking. More men than women show impulsive behavior (71), they experiment earlier with drugs and gambling (72) and are therefore at a greater risk for ending up on the path that could lead to addiction. More women on the other hand, begin taking drugs or gamble as self-medication to reduce stress or alleviate depression (73, 74). The notion that men may gamble for other reasons than women is further in accordance with the Blaszczynski model explaining problematic gambling through three different pathways, the behaviorally conditioned, the emotionally vulnerable and the antisocial-impulsivity pathway (45). More women than men are found to fit in the emotionally vulnerable pathway where psychological distress and anxiety problems are a more common reason for increased gambling (75–77), men on the other hand have found to be more common in the behaviorally conditioned pathway and in the antisocial-impulsivity pathway, where reasons such as excitement and arousal are more typical (36, 45, 78). This leads us to believe that gambling is well-linked to psychiatric comorbidity and possibly more specifically to substance use disorders in both men and women. Psychiatric comorbidity is also common in gambling disorder patients within the health care system, with a higher prevalence of women treated for an affective disorder or an anxiety disorder (79). As introduced in the introduction the “antisocial impulsivist problem gamblers” are a pathway most common for men. On the other hand the emotional vulnerable pathway are more common for women (45). Both pathways support the idea that men and women gamble for reasons such as arousal and stimulation. Men are overrepresented in antisocial impulsivist pathway because they gamble for reasons such as action and arousal and women are overrepresented in the emotional vulnerable pathway because they gamble for reasons to relieve aversive affective states such as anxiety by means of arousal.

Though, more recent studies formulate another picture in where women increase their gambling behavior, the trends and habits of gambling. Women who are born more recently basically appear to be “catching up” with their male counterparts. In Sweden, in one of the largest longitudinal gambling studies conducted so far, there is a clear pattern that the incidence rates between men and women does no longer vary by gender (10). Further, women have been found to have an earlier exposure to gambling and betting halls (80) and along with other countries the online gaming has clearly contributed to an increased gambling behavior in both men and women (10, 12). It has also been argued that online gambling are more addictive than other types of gambling due to its availability and fast speed between bets and wins and are therefore more rewarding than other games (15, 16). However, we did not take this into consideration in our experimental procedure since the action of pressing a button with more force or speed will not impact the slot machine reel to spin faster. This is therefore not correlated to a subjective high or stimulation as was the primary focus of this study. Our findings are also supported by recent pronounced increase of females partaking in gambling behavior which subsequently led to gambling problems compared to men in this group of online gamblers (81). The gambling literature further indicates that both men and women quite equally report a concomitant problem with alcohol or drug disorder, albeit men report larger dependence difficulties compared to women. Female gamblers report uses of alcohol and drugs more frequently compared with female non-gamblers, while male gamblers more often report alcohol and substance dependence compared to male non gamblers (68). Interestingly in a recent publication of an online survey it was found that women with a moderate risk gambling behavior displayed a more severe picture than men did. They reported higher financial problems and feelings of guilt, worse mental health and also in contrast to most previous findings they were as likely as men to require alcohol and drug treatment (81). It has also been seen that the use of a stimulant drug is associated with both gambling frequency and problem gambling in both men and women. High school youths using stimulants such as cocaine or amphetamine six or more times in the past year had a higher likelihood of frequent and at-risk problem gambling behaviors (17). With these studies in mind, the differences in gambling behavior between men and women becomes vaguer. We can only speculate that with an increasing attention to women's gambling behavior, more online choice of gambling opportunities, the concomitant alcohol and drug intake, and the association between psychiatric comorbidity in women the gap between men and women may be less observant.

However, since our main findings stand in contrast to many studies examining gender difference and gambling, a number of limitations deserve to be discussed. Our study participants were thoroughly screened with the SCL-90 (48) for not having any specific psychological problems or gambling addiction problems with the DSM-V (46) and this may be one of the reasons we did not detect any gender differences. Yet, we did observe a few baseline differences in men regarding age, gambling minutes per week and in systolic blood pressure in comparison with women. Men also drank more alcohol units per week than women did although not significantly more. These differences are important to address, and we can only speculate if they may have contributed to our results. In reference to age, both men and women were in their twenties with only a small difference of 4 years. The men drank about two glasses of alcohol more than the women each week and according to their AUDIT scores none of them had any alcohol problems. A difference that is harder to explain is why the men had a significant higher systolic blood pressure than women at baseline. This difference could for example be due to differences in BMI or in stress levels in men and women. The BMI did not demonstrate a higher weight in conjunction with the higher systolic blood pressure that was seen in men. Quite the opposite was seen with a healthy BMI range of around 22 in both men and women.

Our study population was healthy recreational gamblers without any self-reported comorbidities. A laboratory gambling task may have produced a gender difference in a more vulnerable populations such as specific sensation seeking men, psychological distressed women or in subjects that are addicted to gambling. It has for example been seen that men seeking treatment for gambling addiction has found to show a self-reported stronger urge to gamble compared to women (82, 83). Men seeking treatment for drug problems have also been found to have a concomitant gambling problem and antisocial personality disorder in comparison to women (84). Findings of this sort demonstrate that there are gender differences in treatment seeking men with both gambling and drug problems. Furthermore, with regard to the literature, gonadal hormones may account for some individual differences in susceptibility to the reinforcing effects of addictive substances (30, 85), research on gambling tasks and women should be attentive of that gonadal hormone may potentially reduce the self-reported effects. We did not control for menstrual cycle in the previous study in where this *post hoc* analysis is based on (44). Future research including men and women with specific chronic underlying neurobiological disorders, subjects addicted to gambling or studies focusing on gonadal hormones in women may or may not, show more robust gender differences in the subjective effects as a consequence of gambling than what we found.

The time in which the gambling task was presented may also have contributed to our findings. The gambling session in this study was 10 min long and therefore we cannot know if the participant's subjective or physiological responses would have been altered if they had the opportunity to gamble for a longer period of time. The time chosen for the study is to be compared with other studies with a similar approach to ours in where subjective or physiological effects has been found in response to gambling. In a study from Blanchard et al. (86) gamblers in comparison to non-gamblers, were found to show increased heart rates while listening to individualized tapes for 2–3 min. In the study by Carroll and Huxley (87) gamblers were exposed to a slot machine for 5 min finding increased blood pressure in comparison to non-gamblers. In the study by Coventry and Hudson (40) who challenged gamblers with a fruit machine play for 3 min found increased heart rates, and Yucha et al. (43) had their participants gamble for 10 min on a slot machine also found heart rate effects. Based on these studies, most of them seeing effects from the gambling tasks with <10 min of gambling, we expected that our time span would be sufficient to detect differences if there were any. Perhaps we should have had our subjects gamble for a time span in which they individually preferred in minutes.

Another circumstance that may have had an impact on our cardiovascular results is the resting time before measuring blood pressure. We let our participants rest for 10 min before taking the baseline measure. That resting time was based on the recommended minimal time of 3–5 min before taking blood pressure in healthy subjects (88). However, in a more recent study by Mahe et al. (89) it is argued that a minimal of 25 min resting time is needed for a stabilized blood pressure to be taken. The 25 min resting time is foremost described for individuals with hypertension problems however a longer resting time might have created a better baseline for us to detect arousal differences from between men and women. Fluctuations in blood pressure can easily occur in both genders. However, men have a higher baseline blood pressure than women do as a standard measure. This is due to sympathetic and vascular activity that occurs in men but is not seen regularly in young women (90). We did however see an increase in blood pressure and heart rate in both men and women, indicating that our resting time of 10 min before baseline measure was enough to see a significant increase after the gambling task.

Another limitation, and perhaps the most important one, was that the men and women in the study did not gamble enough minutes per week (men gambled for 75 min/week and the females for 22 min/week) for us to detect differences. In a study by Moodie and Finnigan (91) it was found that a frequent gambler that gambled more than 3 times a week showed increased heart rate after playing on a slot machine compared an infrequent gambler who gambled 1–2 times per month. That study did not take gender into account. Perhaps our subjects would have showed differences if they had gambled for a longer period of time each week. This notion is supported by the original study finding that a high gambler that gambles for around 3 h a week showed increased stimulation in comparison to a low or non-gambler that gambles for around 20 min a week (44).

Further, the money our participants gambled with was not real and the stakes were not high. They won an average 350 SEK which is around 38 dollars. It could have been that the stakes were too small to create enough stimulation that may have differed men from women in a considerable way. In a previous study Brown et al. (38) it was found that, in 26 men and 41 women, there were no difference between problem gambler and non-problem gambler in players who had won, but problem gamblers showed greater valence reductions after losing. Unfortunately, we only recorded data on total wins and therefore we cannot know if the outcome would have been different if we would have taken the losses into account. Again, both men and women did similarly self-report rewarding effects of the gambling task even though the stakes were small.

One can also argue that our laboratory gambling task is not a valid analog to a real gambling situation and that much fewer cues are present and therefore not engage or differ men and women as they would in a real setting. The appropriateness of using laboratories, with much less cues, or the real-life setting venues in gambling research has been discussed in an article by Gainsbury and Blaszczynski (92). They found that both settings provide results in the same direction with real life participants sometimes provide less information in response to questionnaires. Based on these results and the above studies presented describing both in and outside the laboratory we believe that the online slot machine in the laboratory that we presented to our subjects was a good analog (38, 40, 42, 43).

This study was a *post hoc* analysis that was not in the first place designed to study gender differences and therefore the results should be interpreted with caution. Our conclusion that there was no difference between men and women stems from a statistically non-significant result, which is not an effective way to prove a hypothesis. However, since the mean and the measures of errors of the self-reported effects were small we do not believe increasing the power in the study would have helped us to detect a difference between men and women.

In conclusion, both men and women showed subjective stimulatory effects of gambling but the effects of the gambling task were not modulated by gender in healthy volunteers. Gambling therefore seems to have the same abuse potential in both men and women. This conclusion is interesting in light of that men have found to be at greater risk for gambling related problems than women. More recently however, women's gambling habits have worsened and therefore the risk of gambling related harm is now more comparable to men. Reporting similar gender results in subjective effects of gambling is important for the understanding of the narrowing gap in men and women's gambling behavior.

## Data Availability Statement

The raw data supporting the conclusions of this article will be made available by the authors, without undue reservation.

## Ethics Statement

All procedures performed in studies involving human participants were in accordance with the Ethical Standards of the Institutional and/or National Research Committee and with the 1964 Helsinki declaration and its later amendments or comparable ethical standards. The patients/participants provided their written informed consent to participate in this study.

## Author Contributions

Both authors listed have made a substantial, direct, and intellectual contribution to the work and approved it for publication.

## Funding

This study was funded by Svenska Spel; Grant number FO2015-0006. This study was supported by the Svenska Spel's Independent Research Council.

## Conflict of Interest

The authors declare that the research was conducted in the absence of any commercial or financial relationships that could be construed as a potential conflict of interest.

## Publisher's Note

All claims expressed in this article are solely those of the authors and do not necessarily represent those of their affiliated organizations, or those of the publisher, the editors and the reviewers. Any product that may be evaluated in this article, or claim that may be made by its manufacturer, is not guaranteed or endorsed by the publisher.
